# The Improvement of the Adaptation Process of Tocopherol and Acetylsalicylic Acid in Offspring of Mothers Exposed to TCDD

**DOI:** 10.3390/ani11123430

**Published:** 2021-12-01

**Authors:** Maciej Dobrzyński, Jan P. Madej, Anna Leśków, Małgorzata Tarnowska, Jacek Majda, Monika Szopa, Andrzej Gamian, Piotr Kuropka

**Affiliations:** 1 Department of Pediatric Dentistry and Preclinical Dentistry, Faculty of Dentistry, Wroclaw Medical University, Krakowska 26, 50-425 Wroclaw, Poland; 2 Department of Histology and Embryology, Faculty of Veterinary Medicine, Wroclaw University of Environmental and Life Sciences, Norwida 25, 50-375 Wroclaw, Poland; jan.madej@upwr.edu.pl (J.P.M.); piotr.kuropka@upwr.edu.pl (P.K.); 3 Department of Basic Sciences, Faculty of Health Sciences, Wroclaw Medical University, Grunwaldzka 2, 50-368 Wroclaw, Poland; malgorzata.tarnowska@umw.edu.pl; 4 Department of Laboratory Diagnostics, 4th Military Hospital, Weigla 5, 50-981 Wroclaw, Poland; jacek_majda@interia.pl; 5 Military Center for Preventive Medicine, Slezna 158, 50-984 Wroclaw, Poland; szopa.monika@wp.pl; 6 Hirszfeld Institute of Immunology and Experimental Therapy, Polish Academy of Sciences, Weigla 12, 53-114 Wroclaw, Poland; andrzej.gamian@hirszfeld.pl

**Keywords:** dioxin, histology, inflammation, antioxidant, adaptation

## Abstract

**Simple Summary:**

Dioxins are proinflammatory factors that may be transferred to offspring through the placenta during pregnancy. α-tocopherol and acetylsalicylic acid are popular agents that limit the spread of inflammation. A histopathological and biochemical analysis was performed to reveal possible changes in liver and blood plasma in response to dioxins, α-tocopherol, and acetylsalicylic acid. The conducted research demonstrated the presence of negative effects on the liver morphology and blood plasma proteins of offspring, due to dioxins that were derived from the mother. However, the use of both drugs can significantly reduce the negative effects on offspring whose mothers have been treated with dioxins.

**Abstract:**

Dioxins are chemical compounds that may cause an inflammatory reaction. During dioxin-induced inflammation, generated reactive oxygen species lead to morphological changes in various tissues and in biochemical parameters. The aim of this study was to demonstrate the changes in the livers of rats whose mothers were exposed to dioxins and the protective role of α-tocopherol and acetylsalicylic acid in liver inflammation. The study material consisted of Buffalo rats who were the offspring of females treated with dioxin, dioxin + α-tocopherol, or dioxin + acetylsalicylic acid. Livers and blood samples were taken from the rats’ offspring, and then histopathological and biochemical analyses were performed. The histopathological analysis showed that the changes observed in the livers of neonates were the result of the dioxins derived from their mother. The biochemical analysis showed that the morphological changes in the liver affected its function, which manifested in a higher total protein concentration in the dioxin-treated group, and that the creatinine level in this group was significantly higher than that in the other groups. This effect was reduced by the protective role of α-tocopherol and acetylsalicylic acid. Based on these results, we came to the conclusion that dioxins significantly affect the structure of the liver, which negatively affects its function, mainly in the scope of the metabolism of plasma proteins and hepatic enzymes.

## 1. Introduction

The liver plays a significant role in inflammatory response, directly affecting the synthesis of acute-phase proteins and the transformation of steroid hormones such as cortisol, estrogens, and testosterone [1,2,3]. It has been proven that there are many factors that affect the ultrastructure of hepatic cells, causing disorders of their secretory function which in turn affects the concentration of acute-phase proteins and the electrophoretic distribution of plasma proteins. Our own studies have shown the significant effect that mechlorethamine and 2,3,7,8-tetrachlorodibenzo-*p*-dioxin (TCDD) have on hepatic metabolism [4,5,6]. In vitro studies have shown that these compounds interfere with the enzyme kinetics of cathepsin b, as well as having the ability to penetrate into lysosomes [7]. The negative effects of dioxins on hepatic metabolism were manifested by the increased frequency and severity of Disseminated Intravascular Coagulation (DIC) in induced pleurisy [8]. It was demonstrated that the dioxins had multiple proinflammatory influences on the organism that consisted of generating free radicals and reactive oxygen species (ROS) through dechlorination, hydroxylation, and epoxidation [9,10]. The negative effects of dioxins on the body were also manifested by the stimulation of cyclooxygenase-2 (COX-2), as well as the induction of Cytochrome P450, family 1, subfamily A, and polypeptide 1 (CYP1A1) synthesis, contributing to increased hydrolase activity, which is responsible for the decomposition of dioxins [11]. It has been shown that the generation of ROS results from the decomposition of dioxins by the enzyme CYP1A1. The mechanism of this process is based on the attachment of the dioxins to an aryl hydrocarbon receptor (AhR) in the hepatocyte cytosol, which leads to the expression of the *cyp1a1* gene and consequently to dechlorination, epoxidation, and hydroxylation [12,13,14]. In response to those processes, there are metabolic disturbances in the liver manifested by hypercholesterolemia, which is associated with increased aspartate transaminase (AST) and alanine transaminase (ALT) levels as well as decreased concentrations of fibrinogen, albumins, and globulins [4,15]. The indirect effect of liver metabolic disorders is manifested in the abnormal degradation of steroid hormones, such as estrogens, testosterone, and cortisol, which are associated with cholesterol metabolism [4,6,16].

Kloser et al. [17] proved that α-tocopherol, in addition to overcoming oxidative stress related to the generation of ROS by dioxins, possesses the blocking properties of an aryl hydrocarbon receptor. Previous studies have shown that when high doses of tocopherol are used in dioxin-contaminated animals, there is a decline in the concentration of diagnostic markers of inflammation and in the results of liver function tests [9,10,18,19]. In addition, it was found that acetylsalicylic acid (ASA) significantly reduces the amount of TCDD binding to cytosolic AhR, as well as potentially blocking the signal transduction initiated by exposure to the dioxin [11,20,21,22]. Studies in type 1-like diabetic rats have indicated that the combination of acetylsalicylic acid and α-tocopherol leads to beneficial changes that could help to protect tissues from thrombotic and ischemic phenomena [23].

A number of our own studies in rats, as well as the observations of other authors [24], have shown that the effects of dioxins are associated with the development of hormonal imbalances, including sex hormones, which affects reproductive functions [25,26,27].

The liver is one of the major organs that is exposed to TCDD due to the high level of metabolism and the immediate proximity of dioxin-accumulating adipose tissue. TCDD and related compounds produce hepatomegaly in all species, even at low doses. Enlarged livers are caused by hyperplasia and the hypertrophy of parenchymal cells, and more specifically by a proliferation of the smooth endoplasmic reticulum [28]. The authors’ own studies have reported that 3 weeks after the administration of 5 μg/kg BW (body weight) of TCDD, macroscopic and histopathological lesions in hepatocytes, manifested by steatosis, were observed in rats [4].

Dioxins have lipophilic properties; hence, they pass from the lipid fraction of plasma to the adipose tissue and liver, as well as passing in the opposite direction. As a result, these compounds are excreted in milk, as found in Eskimo and Japanese women’s milk and polar bear milk [29,30,31]. The consumption of dioxin-contaminated milk resulted in weakened immunity and the occurrence of hermaphroditism in the offspring of polar bears, as well as in microcephaly in children. Fetal and neonatal exposure to dioxins is connected to two routes of transmission from the mother organism—i.e., through the placenta barrier and through breast milk.

The aim of the presented study was to demonstrate the effects of dioxins that were present in the mother organism on her offspring at subsequent stages of development. Due to the fact that the liver is a key organ involved in inflammatory response, the impact of TCDD was assessed on the basis of morphology and the function of this organ, with the simultaneous evaluation of biochemical parameters.

## 2. Materials and Methods

### 2.1. Animals

The study material consisted of rats (Buffalo strain) of both sexes as the offspring of TCDD-treated females. Females were divided into 4 groups, with 6 mothers per group:

− control: not treated with any chemicals;
− TCDD: a single dose of TCDD (2,3,7,8-tetrachlorodibenzo-*p*-dioxin; Greyhound Chromatography and Allied Chemicals, Birkenhead, UK) at a concentration of 5 mg/mL dissolved in dimethyl sulfoxide (DMSO; Sigma-Aldrich, Poznań, Poland) was administered IM (intra-muscular) at a dose of 5 µg/kg BW;
− TCDD and tocopherol (TCDD + E): TCDD in a single dose of 5 µg/kg BW was administered IM and a solution of α-tocopherol acetate (oil solution of the drug prepared individually by Hasco-Lek S.A., Wroclaw, Poland) was administered subdermal once a day for 3 weeks at a dose of 30 mg/kg BW.
− TCDD and acetylsalicylic acid (TCDD + ASA): TCDD at a single dose of 5 µg/kg BW was administered IM and a suspension of ASA in a starch solution (acetylsalicylic acid, Bayer, Berlin, Germany) was administered P.O. (per os) once a day for 3 weeks at a dose of 30 mg/kg BW.

The females were given the above-mentioned compounds for 3 weeks, then they were mated, and labor took place between the 6th and 7th week from the beginning of the experiment. The sampling material was taken from the liver of the offspring at random on the first day after birth (6 samples from each group), in the fourth week after birth (6 samples from each group), and in the sixth week after birth (6 samples from each group). Groups were named as shown in Table 1 below. Blood samples were taken randomly from the rats’ offspring in the sixth week after birth (6 samples from each group).

### 2.2. Histopathological Examination

The liver samples were collected, fixed in 4% buffered formaldehyde (pH 7.4), and routinely processed in paraffin. Sections (6 µm thick) were sliced from each block and stained with hematoxylin and eosin (H&E). The slices were then examined and photographed under a light microscope (Nikon Eclipse 80i; Nikon, Melville, NY, USA) with a video camera.

The severity of the observed pathological changes was estimated using semi-quantitative methods according to the methods found in Klopfleish et al. [32] using our own modifications, where we included additional information. Impaired architectonics, sinusoidal vasodilatation, and hyperemia were evaluated on a 0/1 scale. The hypertrophy of the hepatocytes, multinucleated hepatocytes, hyperchromatic nuclei, foamy cytoplasm, clear or basophilic vacuoles in the hepatocyte cytoplasm, blurred boundaries between hepatocytes, and number of mononuclear cells and eosinophils were evaluated on a scale from 0 to 3 points. The results were summarized for each group and a statistical analysis of the obtained data was performed.

### 2.3. Biochemical Assays

In the sixth week after birth, blood from the rats was collected into standardized hematological and serological test tubes (Sarstedt, Nümbrecht, Germany) while they were under anesthesia, induced by pentobarbital (30 mg/kg BW) into the peritoneum with the use of a cannula catheter injected into the aorta. The electrophoretic separation of blood proteins was performed on an agarose gel according to the manufacturer’s protocol (Beckman Coulter Polska Sp. z o. o., Warsaw, Poland). The reading and analysis of the results were performed at a wavelength of 600 nm using a DT 93 densitometer (Beckman Coulter Polska Sp. z o. o., Warsaw, Poland), according to the manufacturer’s protocol.

The biochemical analysis of the blood collected from the rats was performed on the RA-1000 analyzer (Technikon S.A., Tournai, Belgium) using dedicated reagents purchased from the same manufacturer. Among the measured parameters were:

Total protein (TP)—concentration measured based on the modified biuret reaction in an alkaline environment [33] at λ = 550 nm. Results are given in g/dL. The total precision of the test is ≤2.1% coefficient of variation (CV) and the sensitivity is 1.0 g/l;

Albumin (Alb)—concentration measured using bromocresol green in an acidic environment [34] and absorbance measured at λ = 600 nm. Results are given in g/dL. The total precision of the test is ≤1.4% CV and the sensitivity is 1.0 g/L;

Urea—concentration measured with the use of urease and glutamate dehydrogenase [35] at λ = 340 nm. Results are given in mg/dL. The total precision of the test is ≤2.8% CV and the sensitivity is 1.1 mmol/L;

Creatinine—measurement based on a modified method with picric acid in an alkaline environment [36], with the absorbance measured at λ = 600 nm. Results are given in mg/dL. The total precision of the test is ≤1.7% CV and the sensitivity is 2 μmol/L;

Aspartate aminotransferase (AST)—measured with the use of a Tris-HCl buffer, with L-aspartate and pyridoxal phosphate, based on the International Federation of Clinical Chemistry Protocol [37]. Results are given in U/L. The total precision of the test is ≤2.0% CV and the sensitivity is 2.0 U/L;

Alanine aminotransferase (ALT)—measured based on the IFCC protocol [37]. Results are given in U/L. The total precision of the test is ≤2.5% CV and the sensitivity is 2.0 U/L;

Gamma-glutamyltransferase (GGT)—measured using an automated Konelab 60i biochemical analyzer (ThermoFisher Scientific, Rochester, NY, USA). GGT concentration was provided in U/L.

### 2.4. Statistical Analysis

All obtained data were statistically analyzed with the use of the Statistica v. 9.0 software (Tibco Software Inc, Palo Alto, CA, USA). Means with standard deviations (SD), minimum value ranges (Min), and maximum value ranges (Max) were calculated. The distribution of data was tested with the Student’s t-test. An analysis of variance (ANOVA) and Pearson’s correlation coefficients was calculated to verify the variability of the studied characteristics between the groups. In all analyses, a *p*-value of *p* < 0.05 was considered statistically significant.

## 3. Results

### 3.1. Histopathological Examination

**Figure 1:**
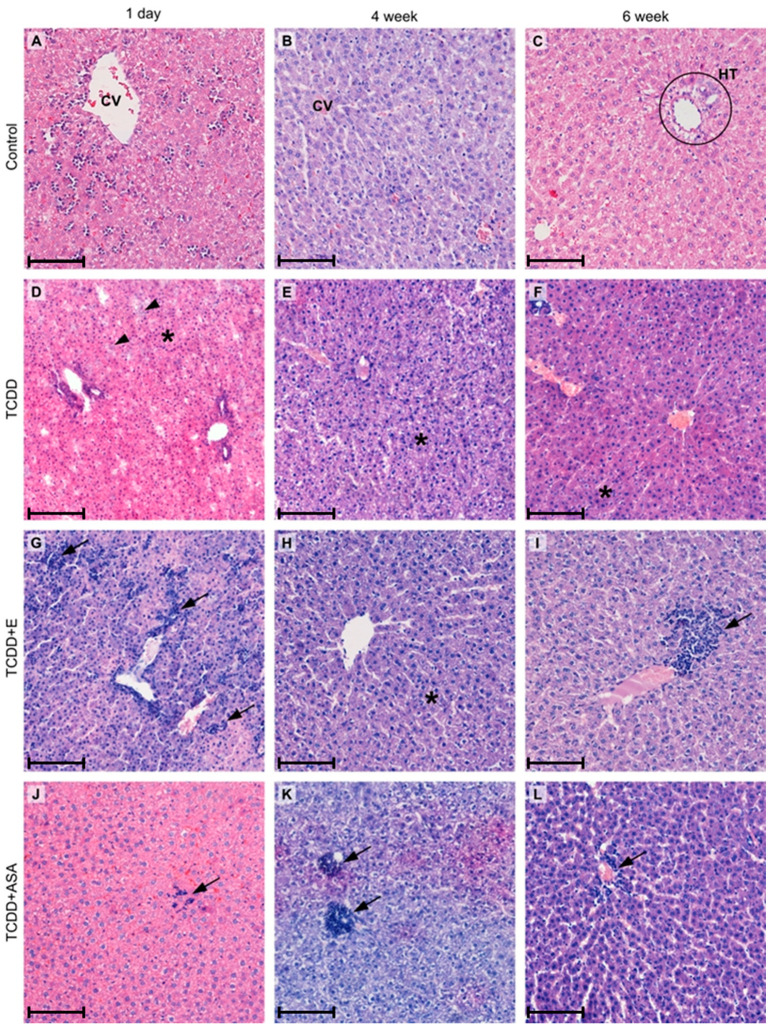
Histopathological changes in the liver of the progeny of TCDD-treated female rats: CV—central vein; HT—hepatic triad; asterisk—impaired architectonics and hepatocytes with hyperchromatic nuclei; arrowhead—basophilic vacuoles in the cytoplasm of hepatocytes; arrow—infiltration of mononuclear cells. Scale bar (**A**–**L**) = 100 µm.

In the group of neonates from the TCDD-treated females, the presence of numerous basophilic vacuoles in the cytoplasm of hepatocytes, the blurring of the intercellular boundaries between hepatocytes, and a disordered liver architecture consisting of the dissociation of hepatocytes were observed (Figure 1). Several liver cells showed a foam structure of the cytoplasm and the hyperchromasia of the nuclei. The presence of single multinucleate cells (polycariocytes) was also observed. In the group of neonates from the TCDD + E-treated females, the predominant symptoms were a very foamy cytoplasm of hepatocytes; the presence of numerous basophilic vacuoles in the cytoplasm; and the occurrence of numerous clusters of white blood cells, mostly eosinophils. The hypertrophy, multinucleation, or hyperchromasia of nuclei were observed in single liver cells.

In the group of 4-week-old rats derived from TCDD-treated mothers, a very foamy cytoplasm with colorless vacuoles, plasmolysis, and hyperchromasia of nuclei were observed in a large number of hepatocytes. In the TCDD + E group, no foamy cytoplasm was observed. An infiltration of white blood cells and single hypertrophic hepatocytes appeared in the preparations. In the TCDD + ASA group, a large number of hepatocytes exhibited a very foamy cytoplasm, as well as plasmolysis and the hyperchromasia of nuclei. Clusters of mononuclear phagocytic cells were also observed, mostly located around the central vein and the hepatic triad. Isolated groups of hypertrophic hepatocytes were also noted.

In the livers of 6-week-old individuals, the hypertrophy of a moderate number of hepatocytes was only observed in the TCDD group. The hyperchromasia of nuclei was observed in all three groups treated with dioxins, but the number of changed cells was highest in the TCDD group, medium in the TCDD + E group, and low in the TCDD + ASA group. Clusters of peripheral blood mononuclear cells (PBMCs) were visible in all groups, but their numbers were higher in TCDD + E and TCDD + ASA, and the latter was accompanied by infiltrations of various cell types in the central vein and the hepatic triad.

**Figure 2:**
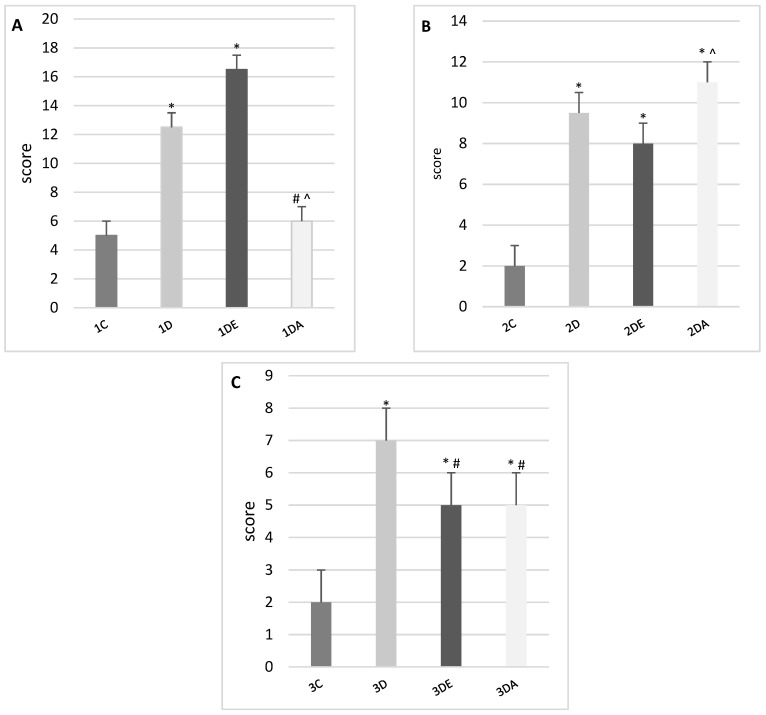
Scoring of histopathological changes in the livers of offspring from TCDD-treated female rats from groups: control, TCDD, TCDD + E, TCDD + ASA (**A**) on the first day after birth, (**B**) four weeks after birth, and (**C**) six weeks after birth. Data are presented as mean ± standard deviation. Statistically significant differences (*p* < 0.05) are marked as follows: * in comparison to control, # in comparison to TCDD group, ^ in comparison to TCDD+E group.

**Figure 3:**
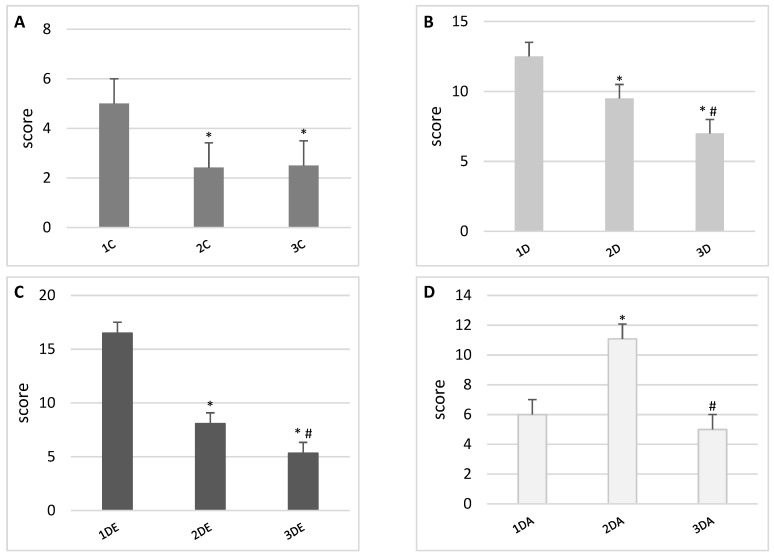
Scoring of histopathological changes in the livers of offspring from TCDD-treated female rats and their progress in time (1—first day of birth; 2—fourth week after birth; 3—sixth week after birth) in groups: (**A**) control, (**B**) TCDD, (**C**) TCDD + E, and (**D**) TCDD + ASA. Data are presented as mean ± standard deviation. Statistically significant differences (*p* < 0.05) are marked as follows: * in comparison to first day of birth, # in comparison to fourth week after birth.

The results of the scoring indicate a gradual improvement in hepatic structure in all the experimental groups (Figure 2 and Figure 3). However, in the ASA group, 4-week-old animals showed a moderate acceleration in cell division (multinucleated hepatocytes) and an increased number of macrophages in the liver. We also observed slight edematic changes in the liver, therefore the scoring revealed some negative pictures of the liver. Liver derived from 6-week-old offspring showed an almost normal structure.

### 3.2. Biochemical Analysis

**Figure 4:**
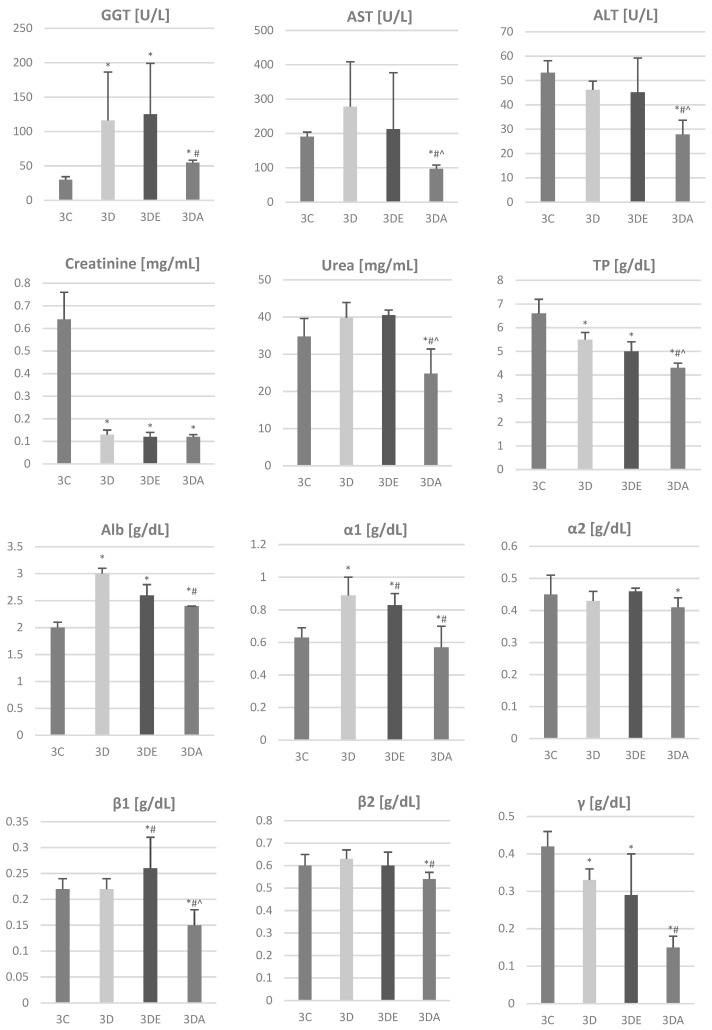
Concentration of selected biochemical markers of liver and kidney function, such as gamma-glutamyltransferase (GGT), aspartate aminotransferase (AST), alanine aminotransferase (ALT), creatinine, urea, total protein (TP), albumin (Alb), globulins (α1, α2, β1, β2, γ) in the blood of rats’ offspring from groups 3C, 3D, 3DE, 3DA. Data are presented as mean ± standard deviation. Statistically significant differences (*p* < 0.05) are marked as follows: * in comparison to control group, # in comparison to TCDD group, ^ in comparison to TCDD + E group.

The results of the biochemical parameters are included in Figure 4. Based on these, the following differences were determined:

GGT levels in the rats in the control group were significantly lower than those in the TCDD group (29.8 vs. 116.0; *p* = 0.039) and TCDD + ASA group (29.8 vs. 125.0; *p* = 0.021). Other differences were statistically insignificant (*p* > 0.05).

Urea levels in the rats in the TCDD + ASA group were significantly lower than those in the rats in the other groups (*p* < 0.01).

Creatinine levels in the rats in the control group were significantly higher than those in the rats in the other groups (*p* < 0.01).

ALT levels in the rats in the TCDD + ASA group were significantly lower than those in the rats in the other groups (*p* < 0.01). Similar correlations were presented in the AST parameter measurement.

Total protein levels in the rats in the TCDD + ASA group were significantly lower than those in the rats in the other groups (*p* < 0.01). Similar correlations were presented in the results of the measurements of concentrations of the α1, β1 of and γ globulins. The level of β2 globulin in the TCDD + ASA group was significantly lower than that in the TCDD group.

## 4. Discussion

The changes that were observed in the livers of neonates probably resulted from the dioxins derived from mother through the placenta. Another possible mechanism, in later periods of development, is that nurslings become affected with dioxins through the transfer of dioxins via milk [38]. Previous studies by other authors have shown that dioxins cause morphological changes in the liver. The affected cells show morphological changes, indicating an increase in endoplasmic reticulum. Moreover, the livers of animals that are chronically subjected to chemicals become fatty. Fat-storing vesicles increase in both size and number [39,40]. The livers of TCDD-exposed mice show an infiltration of inflammatory cells. The liver weight increases by 14% in response to TCDD. These results indicate that the TCDD-exposed mice were free from overt abnormalities in the first 4 days, while liver damage became apparent around day 6 and then progressed. Finally, body weight started to decline around day 14, when the liver damage was clearly manifested [41]. In the studies of Ozeki et al. [22], liver histology showed that TCDD treatment induces a local infiltration of inflammatory cells, and a small number of TUNEL-positive hepatocytes (terminal deoxynucleotidyl transferase-mediated dUTP nick-end -positive) were found only in portions of the pericentral and periportal areas, but not in inflamed regions.

In previous studies by the authors, histopathological changes in the livers of rats treated with TCDD (5 μg/kg BW) were observed, which were manifested through the presence of multiple foci of steatotic hepatocytes (*degeneration adiposa peripherica*), as well as the frequently occurring necrotic foci of these cells. In some animals, a slight hepatic congestion was noted [4,9]. It is significant that indirect effects of dioxins were observed in the offspring of mothers treated with TCDD. In 4-week-old offspring and 6-week-old offspring, morphological changes in the liver were observed, such as foamy cytoplasm with colorless vacuoles as well as the plasmolysis and hyperchromasia of the nuclei.

In the present study, no significant effect of tocopherol was observed on the TCDD-treated mothers in relation to the intensity of histopathological changes in the livers of the neonates. In contrast, a clear impact of tocopherol, manifesting itself as the absence of colorless vacuoles in the cytoplasm of hepatocytes and hepatic cell plasmolysis, was shown in the group of 4-week-old rats as compared to the TCDD group, where these changes were strongly expressed. In the 6-week-old rats, a positive effect of tocopherol and ASA was observed in the absence, or almost complete reduction, of hypertrophic hepatocytes and a visible reduction in the number of cells showing hyperchromasia of the nuclei. This is probably due to the antagonist reaction of the ASA on the aryl hydrocarbon receptor, which is known to induce an inflammation reaction in 2,3,7,8-TCDD-intoxicated organisms [42]. Tocopherol seems to have similar effects, besides its known influence on anti-free radical activity [17].

The previous studies of the authors on the same material have shown that significant changes in the tooth structure during the development of the teeth, as well as in bone mineralization, occurred in all three age groups of rats derived from TCDD-treated mothers [11,25,43].

The studies by Fowler et al. [44] have shown that, after the administration of 5–25 μg/kg BW of dioxins, changes occur in the smooth endoplasmic reticulum of hepatocytes between the sixth and ninth day. These doses of dioxins also contributed to reduced bile secretion and an increased concentration of coproporphyrins [45]. Under the influence of dioxins, rat thymus involution [46], an elevation of corticosteroid levels [47], changes in humoral response, and elevated levels of α- and β-globulins were observed, while delayed immunological reactivity was observed at low doses of TCDD [6,10].

Small changes in the livers of neonates in all groups treated with TCDD may have resulted from the limited metabolic function of this organ during the ontogenic development. During this period of development, toxins are removed from the fetus via the placenta. In the postnatal period, the liver is affected by TCDD from the milk of poisoned mothers and absorbed in the digestive tract of juveniles, which results in pathological changes occurring in 4-week-old and 6-week-old individuals.

The biochemical studies presented in this paper correlate with the described morphological changes in the liver. They indicate that the morphological damage observed mainly in rats whose mothers were treated with TCDD is reflected in biochemical findings. Based on the results, it was found that the level of GGT was statistically significantly higher in the TCDD group and the TCDD + E group compared to the control sample; however, in the TCDD + ASA group the observed increase in this indicator compared to the control group and the significantly lower level than that seen in the TCDD group indicate the protective action of ASA against the TCDD-induced liver damage. A similar correlation of the positive influence of ASA on the changes induced by TCDD can be seen in the results obtained for AST, ALT, urea, and Alb concentration. Morphological lesions were observed in the liver, which resulted in physiological changes that were confirmed by changes in the biochemical and hematological test results [4,6,48,49]. Importantly, a protective effect of vitamin E in rats treated with TCDD and α-tocopherol, which manifested itself in the lack of visible pathological changes in the structure of internal organs and the activity of hepatic enzymes, was also demonstrated [9,10,50].

## 5. Conclusions

The intoxication of offspring by administering 2,3,7,8-tetrachloro-dibenzo-*p*-dioxin to pregnant mothers causes changes in both the liver structure and blood plasma. Dioxins significantly affect the structure of the liver, which negatively affects its function, mainly in the scope of the metabolism of plasma proteins and hepatic enzymes. It has been observed that the administration of acetylsalicylic acid to TCDD-treated mothers results in a reduction in these changes in neonates.

## Figures and Tables

**Table 1:** Codes for experimental groups.

	Control	TCDD	TCDD + E	TCDD + ASA
1st day after birth	1C	1D	1DE	1DA
4th week after birth	2C	2D	2DE	2DA
6th week after birth	3C	3D	3DE	3DA

C—control, D—dioxin, DE—dioxin + E, DA—dioxin + ASA.

## Data Availability

The datasets generated during and/or analyzed during the current study are available from the corresponding author on reasonable request.
